# Ubiquitination and Long Non-coding RNAs Regulate Actin Cytoskeleton Regulators in Cancer Progression

**DOI:** 10.3390/ijms20122997

**Published:** 2019-06-19

**Authors:** Xuda Ma, Yamei Dang, Xiaowen Shao, Xuechun Chen, Fei Wu, Yongmei Li

**Affiliations:** Department of Pathogen Biology, School of Basic Medical Sciences, Tianjin Medical University, Tianjin 300070, China; mxd@tmu.edu.com (X.M.); dangyamei@tmu.edu.com (Y.D.); sxwtmu@gmail.com (X.S.); cxctmu@gmail.com (X.C.); wufei97@tmu.edu.com (F.W.)

**Keywords:** ubiquitination, lncRNA, actin, Rho GTPase, cytoskeleton

## Abstract

Actin filaments are a major component of the cytoskeleton in eukaryotic cells and play an important role in cancer metastasis. Dynamics and reorganization of actin filaments are regulated by numerous regulators, including Rho GTPases, PAKs (p21-activated kinases), ROCKs (Rho-associated coiled-coil containing kinases), LIMKs (LIM domain kinases), and SSH1 (slingshot family protein phosphate 1). Ubiquitination, as a ubiquitous post-transcriptional modification, deceases protein levels of actin cytoskeleton regulatory factors and thereby modulates the actin cytoskeleton. There is increasing evidence showing cytoskeleton regulation by long noncoding RNAs (lncRNAs) in cancer metastasis. However, which E3 ligases are activated for the ubiquitination of actin-cytoskeleton regulators involved in tumor metastasis remains to be fully elucidated. Moreover, it is not clear how lncRNAs influence the expression of actin cytoskeleton regulators. Here, we summarize physiological and pathological mechanisms of lncRNAs and ubiquitination control mediators of actin cytoskeleton regulators which that are involved in tumorigenesis and tumor progression. Finally, we briefly discuss crosstalk between ubiquitination and lncRNA control mediators of actin-cytoskeleton regulators in cancer.

## 1. Introduction

The actin cytoskeleton participates in several basic cellular processes, including cell division, cytokinesis, and motility [1,2,3]. It also plays a prominent role in migration and tumor morphogenesis in cancer cells [4]. Cellular actin exists in two forms: G-actin and F-actin polymers (also referred to as actin filament). All G-actin subunits of F-actin in the same direction; hence, actin filaments are polar. The barbed end of F-actin is more dynamic; polymerization of actin filaments mainly depends on the addition of ATP-bound G-actin to the barbed end, while dissociation is more rapid at the pointed end [5]. The ADF/cofilin family (hereinafter referred to as cofilin) severs and depolymerizes actin filaments and therefore regulates actin filament dynamics and reorganization [5,6,7]. 

Cofilin is inhibited by its phosphorylation and reactivated by its dephosphorylation [8,9]. Phosphorylation at Ser3 of cofilin is the major intersection of actin dynamics and extracellular signals [10,11]. In mammals, kinases of cofilin include LIMKs (LIMK1 and LIMK2), testicular protein kinase 1 (TESK1), TESK2, and Nck-interacting kinase (NIK)-related kinase (NRK)/NIK-like embryo-specific kinase (NESK) [9,12,13,14,15,16]; phosphatases of cofilin include slingshot family protein phosphates (SSHs), including SSH1, SSH2, and SSH3, chronophin, protein phosphate 1, and protein phosphate 2A [7,17,18,19,20,21]. Moreover, cofilin activity is inhibited by binding of phosphatidylinositol 4,5-bisphosphate and cortactin [22,23,24], while it is facilitated by binding of cyclase-associated protein-1 and actin-interacting protein-1 [25,26]. In humans, the Rho GTPase family comprises 20 members, which can be divided into eight subfamilies [27]. The three most important members of the Rho GTPase family, Rac, Rho, and Cdc42, are well-studied upstream regulators of cofilin [4,28]. The p21-activated kinases (PAKs) and Rho-associated coiled-coil containing kinases (ROCKs) are downstream regulators of Rho GTPases and regulate the activity of LIMKs [29,30]. The Rho GTPase protein family and their downstream effectors are under precise control to maintain actin stability. We summarized these regulators of actin cytoskeleton in Table 1.

**Figure 1:**
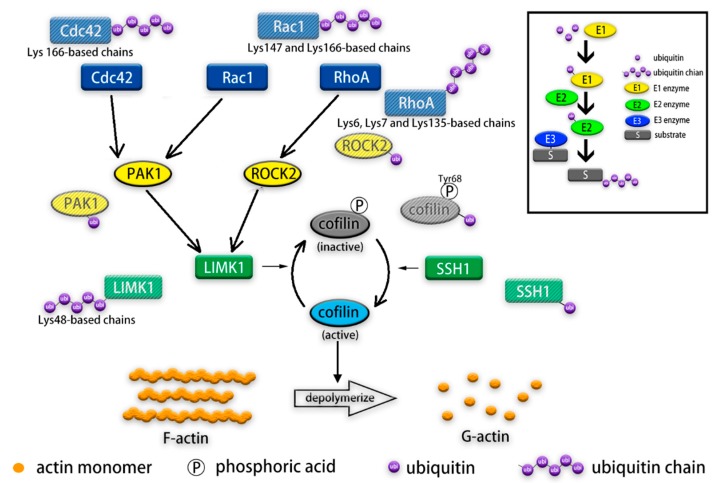
Schematic overview of ubiquitination of actin cytoskeleton regulators. Ubiquitination is a three-step post-transcriptional modification and polyubiquitination of the substrate often leads to its *proteasome-dependent degradation (upper right box). So far, ubiquitination has been found to regulate some actin cytoskeleton regulators, including three well-studied Rho GTPase family members, Rac1, RhoA, and Cdc42, as well as their downstream kinases p21-activated kinase (PAK1) and Rho-associated coiled-coil containing kinase 2 (ROCK2). In addition, cofilin which serves and depolymerizes actin filaments, LIMK1 (cofilin kinase), and SSH1 (cofilin phosphatase) are also regulated by ubiquitination.

Post-translational modification (PTM) like ubiquitination is crucial for activities of actin cytoskeleton-related regulators (Figure 1). Ubiquitin is a highly conserved protein in eukaryotes consisting of 76 amino acid residues. Ubiquitin attaches through its C-terminal glycine to a lysine residue in the target protein as a tag post-translationally and forms an isopeptide bond [80,81,82]. The process of ubiquitin-proteasome system (UPS) degradation begins with activation of ubiquitin by E1 enzymes (or ubiquitin-activating enzymes). The second step involves the transfer of the activated ubiquitin to an E2 enzyme (or ubiquitin-conjugating enzyme). Activated ubiquitin can be transferred to the target protein by three kinds of E3s (also known as ubiquitin ligase): HECT (homologous to E6-AP C-terminus) domain-containing E3s, RBR (RING-between-RING) family E3s and RING (really interesting new gene) finger domain-containing E3s. The former two receive the activated ubiquitin from E2 and subsequently transfers it to the target protein; the latter one catalyzes the transfer of activated ubiquitin from E2 enzyme to the target protein directly [81,83,84,85]. E3 ligase then transfers activated ubiquitin to its substrate, sometimes repeatedly to form polyubiquitin chains. In polyubiquitin chains, monomers may conjugate via several lysine residues or N-terminal methionine residue, producing different ubiquitin signals [86]. Polyubiquitin chains linked via residues such as Lys48 and Lys63 may lead to the proteasome-dependent degradation of the substrate [87].

Long non-coding RNAs (lncRNAs) are RNA molecules without protein coding function that are longer than 200 nucleotides in length. LncRNAs regulate various cellular functions, including actin filament dynamics and reorganization [88]. However, the underlying mechanisms related to the regulation of the actin cytoskeleton and ubiquitination of actin cytoskeleton-related regulators are largely unknown. In this review, we summarize the recent evidence on the ubiquitination of actin cytoskeleton-related regulators (Figure 1), and how lncRNAs regulate ubiquitination of these regulators in cancer progression.

## 2. Ubiquitination of Actin Cytoskeleton Regulators

Ubiquitination can influence the actin cytoskeleton by regulating actin cytoskeleton regulators by different mechanisms. Cofilin phosphorylation can induce its degradation through the ubiquitination pathway. Besides the phosphorylation on Ser3 of cofilin, Tyr68 is phosphorylated by an Src counterpart from a family of tyrosine kinases, v-Src [89]. As a known oncogene, v-Src was found in Rous sarcoma virus. It is commonly activated in colorectal and breast cancers [90]. Phosphorylation on Tyr68 of cofilin increases cofilin ubiquitination thus reducing its activity in stimulating actin depolymerization [89]. 

### 2.1. Ubiquitination and Rho GTPases

As a part of the Ras super family, Rho GTPases are best known for their regulatory functions of cytoskeleton dynamics and many cellular processes including migration, cell polarity, the cell cycle, and cytokinesis [91,92]. Three of all 20 members of Rho GTPase family, RhoA, Rac1, and Cdc42, are the best studied regulators of cofilin.

#### 2.1.1. Ubiquitination of RhoA

Higher levels of active RhoA promote the formation of long unbranched actin filaments in the rear of a migrating cell [93]. RhoA is reported to be ubiquitinated at Lys6, Lys7 and Lys135 by a HECT domain containing E3, Smurf1 (Smad ubiquitination regulatory factor 1) [94,95,96]. Then degradation of RhoA is achieved by Smurf1-mediated proteasome degradation [96]. Smurf1 was first discovered to be recruited by atypical protein kinase C zeta (PKCzeta) in filopodia and lamellipodia, which leads to a rapid membrane extension in response to activation of Cdc42 and Rac1 [94]. Because of its specificity for RhoA, Smurf1 also plays a role in tumor migration and invasion. Smurf1 amplification was found in primary human pancreatic cancers, and overexpression of Smurf1 leads to loss of contact inhibition of NIH-3T3 mouse embryo fibroblast cells [97]. Smurf1 induced degradation of RhoA promotes EGF (epidermal growth factor) induced breast cancer cell migration and invasion [98]. During EMT (epithelial-to-mesenchymal transition), Smurf1-mediated proteasome degradation controls the dissolution of cellular tight junctions under the control of Par6 (partitioning-defective 6), an adapter protein that regulates cellular polarity [95,99]. Smurf1 interacts with a tumor suppressor, RASSF1A (Ras association domain family member 1), to enhance the ubiquitination of RhoA; therefore, RhoA levels are decreased and tumorigenesis is suppressed [100]. The affinities of Smurf1 for RhoA is enhanced by phosphorylation of Smurf1, contributing to increased degradation of RhoA in axon development [101]. RhoA is also ubiquitinated by two members of the F-box protein family, SCF^FBXL19^ (Skp1-Cul1-F-box FBXL19) and SCF^Fbxw7^ (F-box and WD repeat domain-containing7).

SCF^FBXL19^ targets RhoA for ubiquitination and proteasome-dependent degradation in lung epithelial cells [102]. The SCF^FBXL19^-mediated ubiquitination of RhoA is only achieved with phosphorylation of ERK2 (extracellular signal regulated kinase 2). The degradation of RhoA by SCF^FBXL19^ and ERK2 inhibits cell proliferation and stress fiber formation in lung epithelial cells. In gastric cancer cells, SCF^Fbxw7^-mediated downregulation of RhoA induces apoptosis and inhibits EMT [103]. Cul3, a member of the Cullin scaffold protein family, promotes ubiquitination of RhoA through BTB domain-containing adaptors via mediating the assembly of SCF-like ubiquitin ligase complexes [104,105]. One of these complexes, Cul3/BACURD, mediates the polyubiquitination and degradation of RhoA. HeLa cells lacking Cul3/BACURD show less migration potential [106]. 

In addition to proteasome-dependent degradation, autophagy also participates in the degradation of ubiquitinated RhoA. SQSTM1 (sequestosome 1, also known as p62) targets ubiquinated RhoA to the autophagosome [107,108]. Moreover, autophagy selectively degrades GTP-bound RhoA so that the active RhoA maintains an appropriate level at the midbody during cytokinesis [107]. Together with the proteasome, autophagy degradation of ubiquitinated RhoA controls cell motility and genome stability [108]. Interestingly, Smurf1 also participates in the delivery of ubiquitinated RhoA to nascent autophagosomes via its C2 domain [109]. Recently, ubiquitination of the less-understood cytoskeleton regulator RhoB has also been found, as shown in Table 2 [110,111,112].

#### 2.1.2. Ubiquitination of Rac1

So far, several E3s have been reported to target Rac1 for ubiquitination, including HACE1 (HECT domain and ankyrin repeat Containing E3 ubiquitin protein kinase 1), IAPs (Inhibitor of apoptosis proteins), and SCF^FBXL19^. HACE1 preferentially catalyzes polyubiquitination of GTP-bound Rac1 at Lys147 and decreases Rac1 protein levels in breast cancer cells [113]. HACE1 is considered as a tumor suppressor for degradation of Rac1 since HACE1 inhibits reactive oxygen species (ROS) generation by Rac1-dependent NADPH oxidases [119,120,121]. Loss of HACE1 promotes migratory and invasive capabilities of normal mammary epithelial cells MCF12A due to the increased level of activated Rac1 [122]. Under the stimulation of HGF (hepatocyte growth factor), HACE1 increases proteasome degradation of Rac1 and reduces cell migration during epithelial cell scattering [123]. In addition, loss of HACE1 results in excessive levels of activated Rac1 in Xenopus laevis during early embryonic development [124].

IAPs are characterized by the fact they contain at least one Baculovirus IAP Repeat (BIR) domain. IAP proteins are known for their role in the regulation of cell signaling, affecting apoptosis, innate immunity, and tumor shape and migration [114,125,126]. X-linked IAP (XIAP) and cellular IAP1 (cIAP1), which contain a RING domain, function as E3s through binding Rac1 with the RING domains, which leads to the polyubiquitination at Lys147 of Rac1 [114]. Loss of XIAP and cIAP1 leads to stabilization of Rac1 and promotes tumor migration [127]. Interestingly, XIAP has been found to increase endothelial permeability via activation of RhoA, not through its E3 function but through an unknown mechanism [128].

Intriguingly, SCF^FBXL19^ also has ability to target Rac1 for ubiquitination at Lys166 and proteasome-dependent degradation [115]. Unlike its regulation of RhoA, the ubiquitination of Rac1 by SCF^FBXL19^ is accompanied by the phosphorylation of Rac1 by AKT. Induced ubiquitination of Rac1 by SCF^FBXL19^ leads to a reduction in Rac1 levels and decreased cell motility.

#### 2.1.3. Ubiquitination of Cdc42

Unlike RhoA or Rac1, little is known about the ubiquitination of Cdc42. Recently, XIAP was proven to be an E3 ubiquitin ligase of Cdc42 [116]. XIAP directly conjugates ubiquitin chains to the Lys166 residue of the C-terminus of Cdc42 and targets Cdc42 for proteasome-dependent degradation. Mice injected with XIAP-depleted tumor cells have more metastasis nodules in the lung, whereas co-depletion of Cdc42 and XIAP strongly inhibits lung metastasis.

### 2.2. Ubiquitination of PAK1

PAKs are important effectors of Rho GTPase such as Rac1, Cdc42, and Cdc42 homologous protein (Chp, or RhoV) [29,129]. Overexpression of Cdc42 or Chp may paradoxically downregulate PAK1 [130]. Overexpression of Chp reduces PAK1 protein levels via proteasome-dependent degradation, thereby inhibiting T cell chemotaxis, while the related E3 of PAK1 remains unidentified. Ivermectin is a broad-spectrum anti-parasitic drug and a potential anticancer agent. Ivermectin blocks AKT/mTOR signaling and promotes ubiquitination-dependent degradation of PAK1, thereby promoting cytostatic autophagy which inhibits breast cancer [131,132].

### 2.3. Ubiquitination of ROCK

ROCKs are effectors of Rho GTPases, such as RhoA [30]. Downstream effectors of activated ROCKs are involved in processes including actin organization, apoptosis, and development [133]. Little is known about the ubiquitination of ROCKs. ROCK2 can be ubiquitinated by APC/C (anaphase-promoting complex/cyclosome) and induced to degradation to maintain dendritic stability and integrity of neuron cells [117]. APC/C^Cdh1^ is an E3 analog of ROCK2 and works together with its cofactor Cdh1. Thus, APC/C^Cdh1^ might be a potential target against neurodegenerative diseases.

### 2.4. Ubiquitination of LIMKs

LIMKs include LIMK1 and LIMK2; both phosphorylate and inactivate cofilin [134]. The Ring finger protein Rnf6 can ubiquitinate LIMK1 during neuron development leading to its degradation [135]. Rnf6 serves as a ubiquitin ligase and binds to LIMK1, contributing to Lys48-linked polyubiquitination of LIMK1 in the presence of UbcH5, which functions as an E2 enzyme. CRABP2 (cellular retinoic acid binding protein 2) participates in osteogenic differentiation via interacting with LIMK1 in a ubiquitin-proteasome pathway and compromises LIMK1 activity [118]. However, the exact domain of CRABP2 that interacts with LIMK1 and which ubiquitin ligase is involved in this process remain unclear. Parkin is an E3 of LIMK1 and influences activity of LIMK1. In an in vitro experiment, parkin specifically reduces the activity of LIMK1 via ubiquitination in human neuroblastoma-derived BE(2)-M17 cells [136]. No LIMK2 ubiquitination has been found yet.

### 2.5. Ubiquitination of SSH

A relationship between ubiquitin-proteasome dependent degradation and SSHs has rarely been reported. Infection and replication of herpes simplex virus 1 (HSV-1) lead to inactivation of cofilin through ubiquitin-proteasome dependent downregulation of SSH1, which benefits HSV-1 replication in neuronal cells [137].

The known E3s and the ubiquitination sites of actin cytoskeleton regulators are summarized in Table 2.

## 3. LncRNA and the Actin Cytoskeleton

LncRNAs can influence actin directly. For example, the lncRNA CRYBG3 binds G-actin directly, which inhibits F-actin polymerization and blocks cytokinesis of lung cancer cells. The binding also enables lncRNA CRYBG3 to block MAL nuclear localization, thereby inhibiting several immediate early genes which are important in cell proliferation and cancer metastasis [138]. Another lncRNA, TUG1 (taurine up-regulated gene 1), is necessary for EZH2 (enhancer of zeste homolog 2)-mediated methylation of α-actin in rat vascular smooth muscle cells. The formation of the cytoplasmic TUG/EZH2/α-actin complex promotes cortex actin polymerization in synthetic vascular smooth muscle cells [139]. Though lncRNAs exert influence on the actin cytoskeleton of cancer cells in many ways, they mostly act as competitive endogenous RNAs (ceRNAs) for miRNAs by inhibiting miRNAs, thereby increasing expression levels of mRNAs targeted by these miRNAs. Here, we summarized how lncRNAs regulate actin cytoskeleton by interacting with the above-mentioned regulators. Some of these regulators, such as regulators of LIMK, have so far not been found to be controlled by lncRNAs.

### 3.1. LncRNA and Cofilin

The LncRNA GAS5 (growth arrest-specific 5) is currently under intense investigation because of its dysregulation in various diseases, including several types of cancer [140,141,142,143,144], childhood pneumonia [145], and traumatic brain injury [146]. GAS5 is a tumor suppressor that acts by downregulating miR-222 in human glioma cells. In two glioma cell lines, miR-222 knockdown induces upregulation of Plexin C1, which induces cofilin inactivation and thereby promotes cell migration and invasion [147].

### 3.2. LncRNA and Rho GTPases

The regulation of Rho GTPases by lncRNAs has drown much attention, especially in cancer research. Previously, Zou et al. summarized how lncRNAs influence the cytoskeleton by actin regulatory factors during the process of cancer metastasis [88]. Nevertheless, regulation of the cytoskeleton by lncRNAs also occurs in other pathological processes. For instance, the lncRNA NONMMUGO14387 activates the Wnt/PCP pathway after injury during regeneration of peripheral nerve in Schwann cells [148]. Rho family members, such as RhoA and Rac1, are significantly upregulated to promote proliferation and nerve regeneration in Schwann cells. Moreover, the lncRNA LERFS (lowly expressed in rheumatoid fibroblast-like synoviocytes) binds RhoA, Rac1, and Cdc42 mRNA to downregulate their levels under healthy conditions in fibroblast-like synoviocytes. Downregulation of LERFS promotes invasion and migration of fibroblast-like synoviocytes in patients with rheumatoid arthritis [149].

#### 3.2.1. LncRNA and RhoA

Studies have showed that RhoA expression levels are regulated by lncRNAs. Some lncRNAs regulate RhoA expression by acting as ceRNA of specific miRNA, such as the LncRNA XIST (X-inactive-specific transcript) binding to miR-133a-3p under the regulation of CXCR4 (chemokine receptor 4) in an in vitro model of colorectal cancer cells [150]. Other examples are the lncRNA LOC554202, which binds to miR-31 in laryngeal squamous cell carcinoma [151], and lncRNA NORAD (non-coding RNA activated by DNA damage), which binds to miR-125a-3p in pancreatic cancer [152].

There are other mechanisms by which lncRNA regulates on RhoA. Overexpression of the lncRNA PCGEM1 (prostate cancer gene expression marker 1) promotes cancer growth by upregulating protein expression of RhoA and its downstream factors YAP (Yes-associated protein), MMP2 (matrix metalloproteinase 2), Bcl-xL and P70S6K in ovarian cancer [153]. The lncRNA TBILA (TGFβ-induced lncRNA) is upregulated in human germinal center-associated lymphoma and enhances RhoA activation by forming the Smad transcription factor complex [154]. The lncRNA SchLAH (a seven-chromosome locus associated with hepatocellular carcinoma) is lowly expressed in HCC (hepatocellular carcinoma) and downregulates mRNA levels of RhoA and Rac1 in HCC cells [155]. The miRNA miR-31 suppresses breast cancer cell metastasis by targeting genes such as RhoA. Its host gene lncRNA LOC554202 as well as itself are lowly expressed in triple negative breast cancer, due to promoter hypermethylation [156]. The lncRNA AFAP1-AS1 is involved in many types of malignant tumors [157] and upregulates RhoA expression to promote proliferation and metastasis of HCC [158]. MALAT1 (Metastasis associated in lung adenocarcinoma transcript 1) increases protein levels of RhoA and the downstream ROCK in osteosarcoma [159]. Self-assembled TDNs (tetrahedral DNA nanostructures) are degradation-resistant small DNA nanostructures. TDNs promote expression of RhoA, Rac1, and ROCK2 by suppressing the transcription of the lncRNA XLOC010623 and thereby stimulate adipose-derived stem cell migration when internalized by adipose-derived stem cells. TDNs are regarded to have high potential for future applications in regenerative medicine [160].

#### 3.2.2. LncRNA and Rac1

LncRNAs often serve as ceRNAs of Rac1 and promote its expression to promote proliferation and metastasis in various types of cancer, such as MALAT1 binding to miR-509 in osteosarcoma cells [161], the lncRNA TP73-AS1 (TP73 antisense RNA 1) to miR-142 in osteosarcoma cells [162], the lncRNA UCA1 (urothelial cancer associated 1) to miR-126 in myelogenous leukemia cells [163], the lncRNA FTH1P3 (ferritin heavy chain 1 pseudogene 3) to miR-224-5p in uveal melanoma cells [164], and XIST to miR-137 in glioma cells [165].

MALAT1, as mentioned above, promotes the progression of osteosarcoma via upregulation of RhoA, ROCK and Rac1 [161]. Moreover, MALAT1 promotes Rac1 expression as a ceRNA of miR-101b and contributes to liver fibrogenesis in activated hepatic stellate cells [166].

However, several lncRNAs act as tumor-suppressing genes, which are generally lowly expressed in tumor tissue and suppress tumor proliferation or metastasis via repressing Rac1 by unknown mechanisms. Among the reported lncRNAs, we find SchLAH and linc-cdh4-2 in HCC cells [155,167], NBAT-1 in lung cancer cells [168], and MEG3 in primary thyroid cancer [169]. In addition, TUNAR (neural differentiation-associated RNA) represses migration, and invasion of glioma cells by positively regulating miR-200a which suppresses the expression of Rac1 [170].

#### 3.2.3. lncRNA and Cdc42

The lncRNA H19, MALAT1, and SNHG15 (small nucleolar RNA host gene 15) upregulate Cdc42 expression by acting as ceRNAs. H19 targets miR-15b in HCC [171], MALAT1 targets miR-1 in breast cancer cells [172], and SNHG15 binds to miR-153 in glioma vascular endothelial cells [173]. 

The expression levels of Cdc42 are also regulated by lncRNAs. For example, Cdc42 is upregulated upon downregulation of the lncRNA LINC00707 and thereby promotes cell proliferation and migration in lung adenocarcinoma [174]. Overexpression of the lncRNA LINC00339 caused by a genetic variant (rs6426749) on chromosome 1p36.12 suppresses the expression of Cdc42 and elevates the risks of osteoporosis incidence [175]. BDNF-AS (brain-derived neurotrophic factor antisense) plays an inhibitory role in human retinoblastoma. BDNF-AS overexpression leads to cell-cycle arrest and Cdc42 downregulation through a yet unknown mechanism [176].

### 3.3. LncRNA and PAK1

As mentioned above, PAK1 is activated by the lncRNA H19 in HCC cells [171]. H19 is highly expressed in HCC and esophageal squamous cell carcinoma [177,178]. H19 knockdown significantly inhibits EMT, thus providing a new strategy for treating HCC [171].

### 3.4. LncRNA and ROCK

The lncRNA SNHG5 (small nucleolar RNA host gene 5) is dysregulated in several types of cancer [179,180,181]. High levels of SNHG5 function as a sponge for miR-26a and competitively bind miR-26a with ROCK, promoting osteosarcoma cell proliferation, invasion, and migration in osteosarcoma [182].

Table 3 summarizes the above lncRNAs that function as ceRNAs in the regulation of actin cytoskeleton regulators during cancer progression.

## 4. Ubiquitination and lncRNA in Actin Cytoskeleton Regulation

In the last few years, lncRNA regulation of ubiquitination has gradually drawn significant research attention. LncRNAs exert either a positive or negative effect on ubiquitination by various mechanisms. Here, we briefly summarize these mechanisms as follows. (1) LncRNA acts as ceRNA for miRNA of ligases and promotes expression of these E3s [183,184,185,186,187,188]. For example, MALAT1 promotes FBXW7 expression by acting as ceRNA for miR-155 in glioma cells [185]. The regulations of ubiquitination by lncRNAs of actin cytoskeleton regulators occurs via these means. (2) LncRNA binds to the substrate and blocks its interaction with E3 [189,190,191]. For instance, the lncRNA HOTAIR binds with the androgen receptor and blocks its binding to E3 MDM2 (mouse double minute 2) [189]. (3) LncRNA binds to E3. LincRNA-p21 enhances the activity of p53 by directly binding MDM2. Intriguingly, lincRNA-p21 is also a transcriptional target of p53, which forms positive feedback that promotes the activity of p53 [192]. (4) LncRNA enhances the interaction between E3 and its target [193]. For example, the lncRNA CCDST (cervical cancer DExH-box helicase 9 suppressive transcript) acts as a scaffold between DHX9 (DExH-box helicase 9) and MDM2 to promote the degradation of DHX9 [193]. (5) LncRNA directly links the substrate to UPS components [194]. The lncRNA NRON inhibits HIV-1 replication by directly attaching viral transactivator protein Tat to Cul4B and PSMD11 (proteasome 26S subunit) and promotes the degradation of Tat [194]. (6) LncRNA recruits deubiquitinase and inhibits ubiquitination [195]. The lncRNA LINC00473 inhibits ubiquitination by recruitment of deubiquitinase USP9X and thereby promotes proliferation and invasion of HCC cells [195]. (7) LncRNA regulates ubiquitination via other PTMs of the target protein, such as methylation [196] and phosphorylation [197]. Phosphorylation of β-catenin leads to its ubiquitination and degradation. Its E3 ligase βTrCP catalyzes the ubiquitination of β-catenin at K19 and K49. Lnc-b-Catm, which is highly expressed in liver cancer stem cells and recruits EZH2 (enhancer of zeste 2 polycomb repressive complex 2 subunit) to directly catalyze K49 methylation of β-catenin, inhibits the phosphorylation and subsequent ubiquitination of β-catenin [196]. The lncRNA MEG3 promotes the phosphorylation of EZH2 at Thr-345 and Thr-487, and consequently lowers protein levels of EZH2 by promoting its ubiquitination and degradation [197]. Therefore, lncRNAs play an essential role in ubiquitination and proteasome-dependent protein degradation. Understanding how lncRNAs regulate ubiquitination helps us understand how lncRNAs are involved in cancer progression. Meanwhile, based on these mechanisms, the lncRNAs provide potential therapeutic targets for multiple cancer treatment strategies. 

So far, several E3s that affect the actin filament regulation pathway have been proven to be controlled by lncRNAs. For instance, SCF^FBXW7^ and SCF^FBXL19^ are regulated by lncRNAs. The relatively well-studied FBXW7 belongs to the F-box protein, which are part of the SCF family ligase complexes. SCF^FBXW7^ is regarded as a tumor suppressor of multiple human cancers because it degrades several proto-oncogenes, Notch and Myc [198]. The lncRNA MIF (c-Myc inhibitory factor) is a ceRNA for miR-586 and increases FBXW7 levels, which subsequently promotes c-Myc degradation and inhibits aerobic glycolysis and tumor progression [183]. It is noteworthy that a feedback loop exists between MIF and c-Myc; overexpression of c-Myc induces transcription of MIF. The lncRNA MT1JP (metallothionein 1J, pseudogene) increases expression levels of FBXW7 and inhibits proliferation and invasion of gastric cancer cells [199]. Moreover, MT1JP regulates FBXW7 and inhibits gastric cancer via binding to miR-92a-3p [184]. MALAT1 also promotes FBXW7 expression by acting as a sponge of miR-155 in glioma cells [185]. The lncRNA TINCR (Terminal differentiation-induced lncRNA) suppresses proliferation and invasion of lung cancer cells by serving as a ceRNA to miR-544a and upregulating FBXW7 [186]. The lncRNA CASC2 (cancer susceptibility candidate 2) acts as a sponge of miR-367 and upregulates FBXW7, thereby inhibits EMT in HCC cells [187]. Intriguingly, although there is currently no evidence that lncRNA interacts with FBXL19, an antisense transcript of FBXL19, FBXL19-AS1, was previously discovered to be oncogenic in colorectal cancer and osteosarcoma [200,201]. FBXL19-AS1 acts as a sponge of miRNA in cancer; however, whether there is any pathological or physiological interaction between FBXL19-AS1 and FBXL19 mRNA still needs further study. The lncRNA SPRIGHTLY promotes cellular proliferation in melanoma cells. XIAP, targeting Rac1 for proteasomal degradation, is significantly upregulated in SPRIGHTLY-overexpressing melanocytes [188].

Theoretically, lncRNAs may be capable of regulating proteins involved in the ubiquitination process, while ubiquitination directly modifies proteins but not RNAs. Thus, lncRNA is likely to be regulated by ubiquitination via ubiquitination-dependent degradation of upstream transcription factors or proteins that interact with lncRNA. However, little research on this mechanism has been published so far.

## 5. Conclusions

The actin cytoskeleton plays an important role in modulating cell motility and cell morphology, thus the regulation of actin regulatory factors is crucial to normal cell function. Though various diseases are related to this topic, most present research focuses on this regulation in neuron diseases and, especially, tumor progression and metastasis. As is well known, ubiquitination-dependent degradation is a major mechanism by which appropriate cellular protein levels are maintained. It is easy to understand that abnormal ubiquitination of the actin cytoskeleton regulators may lead to higher metastasis potentiality, but it is more likely to lead to tumor inhibition. For example, the E3 ligase HACE1 is a tumor suppressor in natural killer cell malignancies and breast cancer [116].

Ubiquitination is one of the best understood PTMs. Nevertheless, some questions remain unanswered. For instance, ubiquitination of several regulators has been established, and its role in the progression of certain diseases has been confirmed, but the enzymes involved in the ubiquitination of some particular regulators remain to be identified. Much research has focused on the functions of lncRNAs because of their simultaneous participation in multiple steps of cancer progression or simultaneous involvement in multiple types of cancer, as is the case for MALAT1 [161,172,173,185]. As mentioned above, lncRNAs can affect ubiquitination by many mechanisms. However, lncRNAs often act as ceRNA and increase certain protein level to change actin cytoskeleton in cancer cells (Table 3), which indicates that this mechanism may widely exist. Interestingly, E3s, like MDM2, may be regulated by different lncRNAs in various ways [189,192,193]. Whether these lncRNAs are related or competing with each other is also needed to be elucidated. In this review, we briefly discussed how lncRNA regulates the actin cytoskeleton via interaction with ubiquitination processes. Ubiquitination and lncRNA regulation of these regulators are essential and require further study. Further research can help us understand more about these diseases and open possibilities for clinical applications.

## Figures and Tables

**Table 1:** Cellular structures regulated by actin cytoskeleton regulatory factors.

	Rho GTPases			PAKs		ROCKs	LIMKs	SSH1
	RhoA	Rac1	Cdc42	PAK1	PAK4			
**podosomes**	−: [31,32]	+: [33,34]	+: [35,36]	+: [37,38]	+: [39]	−: [40,41]	\	\
**invadopodia**	−: [42,43] +: [44,45,46]	−: [47,48] +: [42,49]	+: [46,49,50,51]	−: [48]	+: [52]	+: [53,54,55]	+: [53,54,55,56,57]	\
**filopodia**	−: [58,59] +: [60,61,62,63]	+: [64,65,66]	+: [28,67,68,69]	+: [66,70]	\	−: [59,71] +: [61,63]	+: [63]	\
**lamellipodia**	+: [62,72]	+: [28,73]	+: [28,73,74]	+: [75,76]	\	+: [72]	+: [76,77,78]	+: [77,79]

Numbers refer to references; −: inhibition; +: promotion; \: unknown.

**Table 2:** E3s and ubiquitination sites of actin cytoskeleton regulators.

Regulator		Ubiquitination Site	E3 Ligase	Reference
**Rho GTPases**	**RhoA**	Lys6, 7 and 135	Smurf1	[94,95,96]
		\	SCF^FBXL19^	[102]
		\	SCF^FBXW7^	[103]
		\	Cul3/BACURD	[106]
	**RhoB**	Lys162 and Lys181	CUL3/KCTD10	[110,111]
		Lys6 and Lys7	Smurf1	[112]
	**Rac1**	Lys147	HACE1, IAPs	[113,114]
		Lys166	SCF^FBXL19^	[115]
	**Cdc42**	Lys166	XIAP	[116]
**ROCK**		\	APC/CCdh1	[117]
**LIMK1**		Lys48	Rnf6	[118]

**Table 3:** Long non-coding RNAs (lncRNAs) as competitive endogenous RNAs (ceRNAs) of miRNAs in regulation of the actin cytoskeleton.

		LncRNA	miRNA	Cancer	Reference
**Rho GTPases**	**RhoA**	XIST	miR-133a-3p	colorectal cancer	[150]
		LOC554202	miR-31	laryngeal squamous cell carcinoma	[151]
		NORAD	miR-125a-3p	pancreatic cancer	[152]
	**Rac1**	MALAT1	miR-509	Osteosarcoma	[161]
		TP73-AS1	miR-142	Osteosarcoma	[162]
		UCA1	miR-126	myelogenous leukemia	[163]
		FTH1P3	miR-224-5p	uveal melanoma	[164]
		XIST	miR-137	Glioma	[165]
	**Cdc42**	H19	miR-15b	hepatocellular carcinoma	[171]
		MALAT1	miR-1	breast cancer	[172]
		SNHG15	miR-153	Glioma	[173]
**PAK1**		H19	miR-15b	hepatocellular carcinoma	[171]
**ROCK**		SNHG5	miR-26a	Osteosarcoma	[182]
**COFILIN**		GAS5	miR-222	Glioma	[147]

## Abbreviations

ADF	actin-depolymerizing factor
Ser3	serine residue at position 3
TESK	testicular protein kinase
NIK	Nck-interacting kinase
NRK	Nck-interacting kinase related kinase
NESK	Nck-interacting kinase related kinase/NIK-like embryo-specific kinase
SSH	slingshot family protein phosphate
UPS	ubiquitin-proteasome system
HECT	homologous to E6-AP C-terminus
RBR	RING-between-RING
RINGCRABP2	Cellular retinoic acid binding protein 2
PAK	p21-activated kinase
ROCK	Rho-associated coiled-coil containing kinase
APC/C	anaphase-promoting complex/cyclosome
APC/C^Cdh1^	APC/C with its cofactor Cdh1
HSV-1	herpes simplex virus 1
Smurf1	Smad ubiquitination regulatory factor 1
PKCzeta	protein kinase C zeta
EMT	epithelial-to-mesenchymal transition
Par6	partitioning-defective 6
RASSF1A	Ras association domain family member 1
SCF	skp1-cul1-f-box
SCF^FBXL19^	Skp1-Cul1-F-box FBXL19
FBXL19	F-box and leucine rich repeat protein 19
Erk2	extracellular signal regulated kinase 2
Fbxw7	F-box and WD repeat domain-containing7
SCF^Fbxw7^	Skp1-Cul1-F-box Fbxw7
SQSTM1	sequestosome 1, also known as p62
HACE1	HECT domain and ankyrin repeat Containing E3 ubiquitin protein kinase 1
IAP	Inhibitor of apoptosis protein
ROS	reactive oxygen species
HGF	hepatocyte growth factor
BIR	Baculovirus IAP Repeat
XIAP	X-linked IAP
cIAP1	cellular IAP1
TUG1	taurine up-regulated gene 1
EZH2	enhancer of zeste homolog 2
ceRNA	competitive endogenous RNAs
GAS5	growth arrest-specific 5
LERFS	lowly expressed in rheumatoid fibroblast-like synoviocytes
XIST	X-inactive-specific transcript
CXCR4	chemokine receptor 4
NORAD	non-coding RNA activated by DNA damage
PCGEM1	prostate cancer gene expression marker 1
TBILA	TGFβ-induced lncRNA
SchLAH	seven chromosome locus associated with hepatocellular carcinoma
HCC	hepatocellular carcinoma
MALAT1	metastasis associated in lung adenocarcinoma transcript 1
TDNs	tetrahedral DNA nanostructures
UCA1	urothelial cancer associated 1
FTH1P3	ferritin heavy chain 1 pseudogene 3
TUNAR	neural differentiation-associated RNA
SNHG15	small nucleolar RNA host gene 15
BDNF-AS	brain-derived neurotrophic factor antisense
SNHG5	small nucleolar RNA host gene 5
MIF	c-Myc inhibitory factor
CCDST	cervical cancer DExH-box helicase 9 suppressive transcript
DHX9	DExH-box helicase 9
MDM2	mouse double minute 2
PSMD11	proteasome 26S subunit
MT1JP	metallothionein 1J, pseudogene
TINCR	Terminal differentiation-induced lncRNA
CASC2	cancer susceptibility candidate 2
